# New *Bythinella* (Gastropoda, Bythinellidae) species from western Turkey

**DOI:** 10.3897/zookeys.481.8225

**Published:** 2015-02-04

**Authors:** Mehmet Zeki Yıldırım, Ümit Kebapçı, Seval Bahadır Koca, Arzu Yüce

**Affiliations:** 1 Faculty of Education, Mehmet Akif Ersoy University, Burdur, Turkey; 2 Faculty of Arts and Sciences, Mehmet Akif Ersoy University, Burdur, Turkey; 3 Faculty of Fisheries, Süleyman Demirel University, Eğirdir, Isparta, Turkey; 4 Kocaeli University, Hereke O.I. Uzunyol Vocational School, Kocaeli, Turkey

**Keywords:** *Bythinella*, new species, freshwater, springs, Turkey

## Abstract

*Bythinella anatolica* **sp. n.**, *Bythinella istanbulensis* **sp. n.**, *Bythinella magdalenae* **sp. n.**, and *Bythinella wilkei* **sp. n.** from western Turkey are described herein. Illustrations of the shell and genitalia of the newly described taxa, together with comparisons with previously known *Bythinella* taxa and a key to the species from western Turkey, are also provided.

## Introduction

*Bythinella* Moquin-Tandon, 1856, the sole genus of the caenogastropod family Bythinellidae (Szarowska 2006; Wilke et al. 2013), is composed of small sized (1–3 mm) species occurring almost exclusively in springs (rarely in upper courses of nutrient poor montane streams or caves) having relatively cold waters below 10 °C (Boeters 1998). Although typically characterized by cylindrical (sometimes ovate-conic) shells with rounded apertures, congeners are difficult to discriminate owing to intraspecific variation and the morphostatic mode of divergence observed in the genus (Falniowski et al. 2012). The genitalia of these snails is characterized by a penial appendix with a flagellum in the male (Glöer and Pesic 2010), and a J-shaped cylindrical bursa copulatrix in the female (Falniowski et al. 2009b).

*Bythinella* contains 132 species and subspecies (Yıldırım et al. 2006; Georgiev 2009; Glöer and Georgiev 2009; Bank 2013; Georgiev and Glöer 2013; Glöer 2013; Odabaşı and Georgiev 2014; Georgiev and Glöer 2014; Glöer and Pešić 2014) and is among the most species-rich genera in the Truncatelloidea. The geographic range of the genus extends from northern Africa and the Iberian Peninsula through central Europe to the Balkan countries, Ukraine and Turkey (Kristensen 1985; Haase et al. 2007). Until recently, only a few species had been recorded in the eastern half of this range. All 21 of the species in Bulgaria and 10 of 12 species in Romania were described within the last decade (Falniowski et al. 2009a, b; Georgiev and Glöer 2013, 2014); although only three species are known from continental Greece (Bank 2013), the actual number is estimated to be 10 based on molecular data (Falniowski and Szarowska 2011). Together with difficulties in conchological identification mentioned above, the small number of local researchers and scant material from the area have contributed to the recognition of only a few species of *Bythinella* in its eastern range until recently. The case in Turkey, forming the eastern limit for the genus, does not differ much in this regard. The first mention of the genus from this country was by Schütt (1965), who reported *Bythinella opaca* (Frauenfeld, 1857) from Belgrad Forest in Prov. Istanbul. The assignment of the species from Istanbul to *Bythinella opaca* (Frauenfeld, 1857) was later refuted by Schütt (1980) and Yıldırım et al. (2006), however the taxonomic status of this snail remains uncertain. Three additional species from the Asian part of Turkey have also been described (Kebapçı and Yıldırım 2010; Odabaşı and Georgiev 2014): *Bythinella turca* Radoman, 1976 from Cire spring (Prov. Isparta), *Bythinella occasiuncula* Boeters & Falkner, 2001 from Kırkoluk springs (Prov. İzmir), and recently described *Bythinella kazdaghensis* Odabaşı & Georgiev, 2014 from Ayazma Stream (Prov. Çanakkale).

In the present study, four new *Bythinella* species are described from western Turkey based on field surveys between 2006 and 2013. These species are discriminated based on details of shell morphometry, head and cephalic tentacle pigmentation, penial appendix, tubular gland, female genitalia, central tooth of radula and operculum. A comparison with other species and an identification key to other congeners described from Turkey are also presented.

## Material and methods

Specimens were collected by hand-netting and preserved in 75% ethanol. Dissections and measurements of the genital organs and the shells were carried out using a Olympus SZ12 stereo microscope; photographs were taken with a digital camera system. Morphological terminology largely follows Radoman (1973, 1976) and Hershler and Ponder (1998). The scale bars in the figures are 1 mm.

Abbreviations: SW: shell width, SH: shell height, rs: reseptaculum seminis, ah: aperture height, aw: aperture width, Coll. Yıldırım: Collection of M. Zeki Yıldırım in Zoological Museum of the Mehmet Akif University (Burdur, Turkey).

## Systematics

### Genus Bythinella

Taxon classificationAnimaliaLittorinimorphaBythinellidae

Moquin-Tandon, 1856

#### Type species.

*Bulimus viridis* Poiret, 1801.

### Bythinella anatolica

Taxon classificationAnimaliaLittorinimorphaBythinellidae

Yıldırım, Kebapçı & Bahadır Koca sp. n.

http://zoobank.org/F4B75E78-F447-40C7-BCF0-5B2FA8D00A57

Figs 1 , 2 , 6a

#### Holotype

(Coll. Yıldırım): SH 2.78 mm, SW 1.67 mm, ah 1.22 mm, aw 1.11 mm. Coll. Yıldırım; TURKEY, Manisa, Çırpıcıdede hill on Spil Mountain, N 38°44.66', E 27°24.30', 17. 07. 2006. Leg. M. Z. Yıldırım. Paratypes: 18 ex. (5 dissected), same data and locality as holotype in Coll. Yıldırım.

#### Type locality.

A spring below Çırpıcıdede hill on Spil Mountain and its small outflow stream down the hill, Manisa.

#### Etymology.

Named after Anatolia.

#### Description.

Shell thin, usually cylindrical-ovoid (SW/SH 57.19%), appearing blackish (owing to darkly pigmented animal), with slightly rough surface; apex blunt, depressed on the left side; having 3 ^1^/_2_–4 very tumid whorls (more convex the left side), last whorl more inflated, sutures deep. Aperture ovoid or pear shaped, height of the aperture usually greater than penultimate whorl, umbilicus relatively broad and deep, sometimes covered by the outer lip, palatal lip margin not reflected, columellar and parietal margins broadly reflected.

Head black; tentacles unpigmented. Operculum oval and with distinct growth lines.

**Figure 1.**
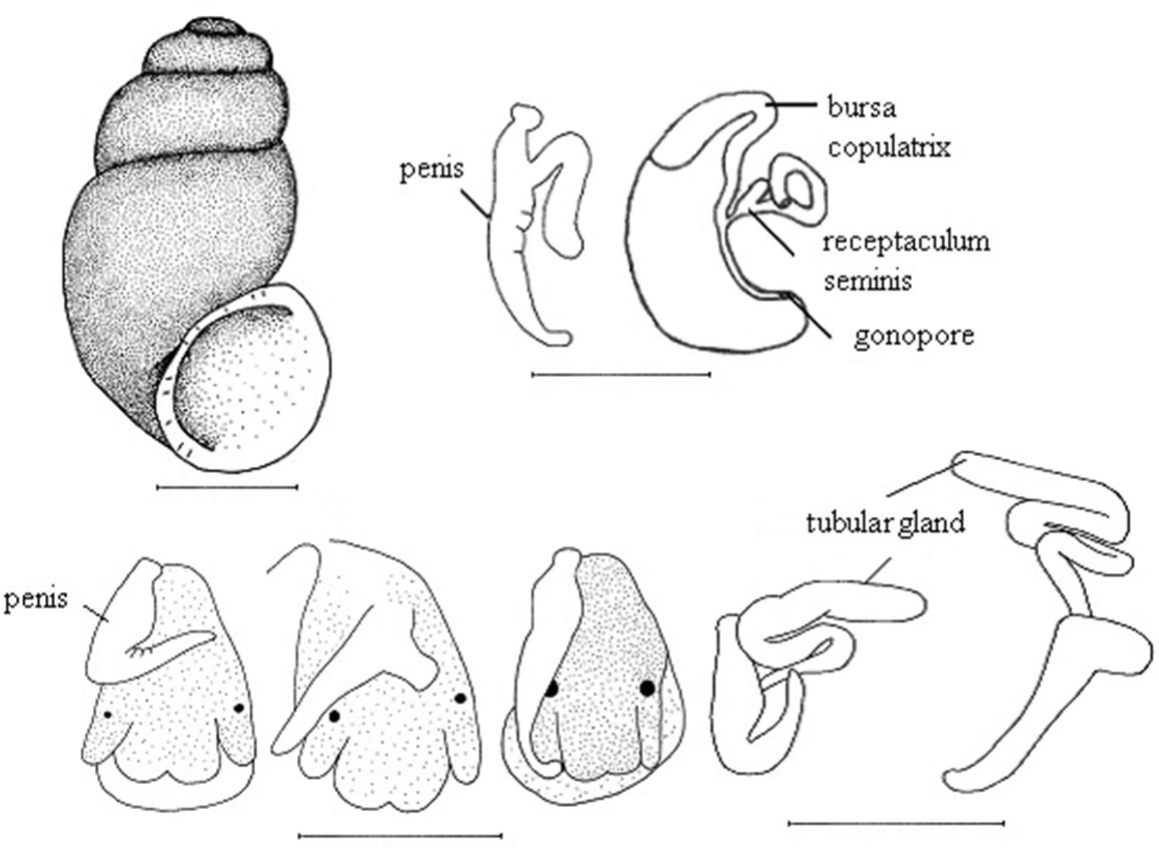
Shell and anatomy of a *Bythinella* species (*Bythinella anatolica* sp. n.). Scale bars = 1 mm.

**Figure 2.**
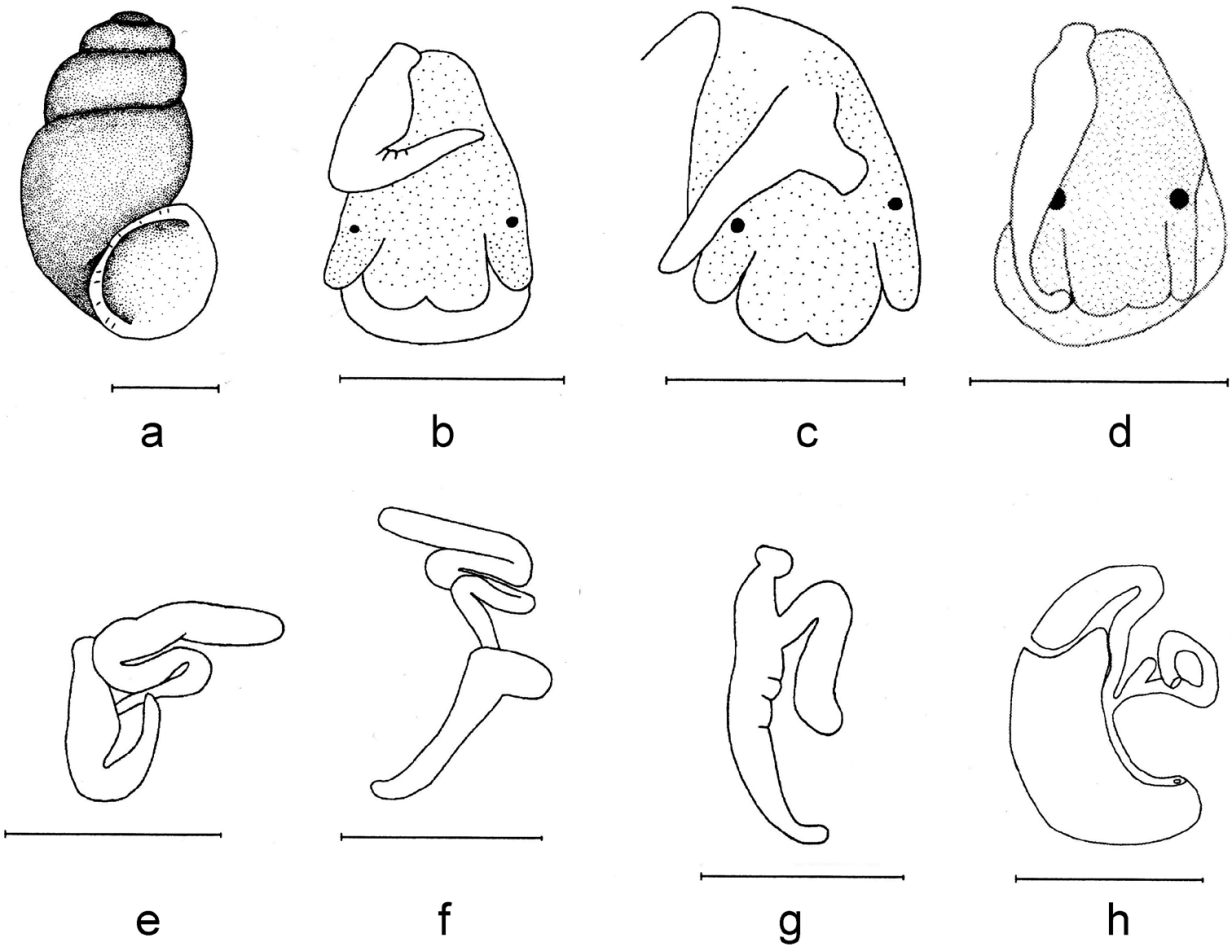
Shell and anatomy of *Bythinella anatolica* sp. n.: **a** shell **b, c, d** male head and various positions of penis **e, f, g** penes **h** female genitalia. Scale bars = 1 mm.

Penis unpigmented and variable in natural position among individuals (straight to bent or folded), though not in shape. Tubular gland thickened, penial appendix usually very short (varying according to the shell size of the individuals). Bursa copulatrix narrow and elongated, rs_1_ large and globular in shape. Central tooth of radula with 9 pointed cusps, 1 median and 4 each on sides; lateral margin without any cusps, but undulated; basal lip roundish (Figures 1, 2).

Measurements (n=19): SH: 2.79 mm (min 2.37/max 3.07), SW: 1.65 mm (min 1.37/max 1.92), SH/SW: 1.7 (min 1.59/max 1. 91), SW/SH: 0.58 (min 0.52/max 0.62), ah/SH: 0.44 (min 0.41/max 0.46).

Differential diagnosis: Identified by its larger shell (except *Bythinella wilkei* sp. n.) and ear–like aperture having reflected margins, a character state not observed in other Turkish species.

#### Habitat.

Specimens were collected from under the stones in the spring outflow down the hill.

#### Remarks.

The new species is distinguished from other Turkish species by its larger shell dimensions and ear like shell aperture. *Bythinella turca* Radoman 1976 is distinguished from *Bythinella anatolica* sp. n. by its blunter and shorter shell (SH 2,42 ± 0,13; max. 2.81 mm). SW/SH is close to *Bythinella istanbulensis* sp. n., from which it can be distinguished by its oval shell with convex whorls. *Bythinella occasiuncula* and *Bythinella magdalenae*, the geographically most proximate congeners, have smaller shell dimensions, while *Bythinella occasiuncula* can be identified by the shorter and blunter shell shape and *Bythinella magdalenae* sp. n. by smoother periphery and shallow sutures.

According to our current knowledge of Peri-Aegean *Bythinella* species, the area of the Aegean coast of Turkey is a center of diversity for the genus. The Eastern Aegean Islands Ikaria, Kos, Lesbos, and Chios are inhabited by *Bythinella kosensis* (Schütt 1980; Bank 1988), while within the provinces of Aydın, İzmir, and Manisa on adjacent Anatolian mainland there are three species (*Bythinella anatolica* sp. n., *Bythinella magdalenae* sp. n. and *Bythinella occasiuncula*) geographically isolated from the remainder of the species recorded in Turkey (Figure 7).

### Bythinella istanbulensis

Taxon classificationAnimaliaLittorinimorphaBythinellidae

Yıldırım, Kebapçı & Yüce sp. n.

http://zoobank.org/8958B919-4F97-4EAA-A6F8-EB7059A8FEB7

Figs 3 , 6b

Bythinella opaca , Schütt, H., 1965 Zur Systematik und Ökologie Türkischer Süsswasserprosobranchier. Zoologische Mededelingen, 41: 43–71. (misidentification).
Bythinella “opaca” , Schütt, H., 1980 Zur Kenntnis griechischer Hydrobiiden, Arch.Moll.110 (4/6):115.
Bythinella sp. A, Yıldırım et al. 2006 Supplement to the Prosobranchia (Mollusca: Gastropoda) Fauna of Fresh and Brackish Waters of Turkey, Tr. J. Zool. 30: 197–204.

#### Holotype

(Coll. Yıldırım): Shell height: 2.74 mm, width 1.43 mm (Coll. M.Z. Yıldırım); TURKEY, Istanbul, Bahçeköy, a small spring at the entrance of Bahçeköy in Belgrad Forest, N41°11.09', E28°59.5', 23.02.2013, leg. A. Yüce. Paratypes: 27 ex., same data and locality as holotype; 5 ex. Zoologisches Museum Hamburg (ZMH 79661), 5 ex. Naturhistorisches Museum in Wien (NHMW 109174), rest in the Coll. Yıldırım. Additional material: 5 ex. in the University of Giessen (Coll. Prof. Wilke), rest in the Coll. Yıldırım (5 dissected).

#### Type locality.

A small spring at the entrance of Bahçeköy in Belgrad Forest, Bahçeköy, Istanbul.

#### Etymology.

Named after the type locality.

#### Description.

Shell broad cylindrical oval, yellowish horn-colored, surface smooth and glossy covered by a thick, solid and rough encrustation due to chemical conditions of the spring water; apex blunt; 3½–4 convex whorls, rapidly and regularly increasing to form a cylindrical shape, last whorl about ^2^/_3_ of the shell height, sutures relatively deep; aperture pear-shaped, height nearly the same as or slightly less than the penultimate whorl, columellar margin with an distinct inner lip reaching basal and apical corners of the aperture; operculum orange, umbilicus narrow and slit-shaped (Figs 2a, 6a).

Tubular gland relatively short and thick. Pallial roof unpigmented, head having little pigmentation (Fig. 2b), rs_1_ small and attached to oviduct (Fig. 2c), thus not easily discernible.

Measurements (n=28): SH: 2.67 mm (min 2.18/max 2.87), SW: 1.66 mm (min 1.37/max 1.94), SH/SW: 1.61 (min 1.39/max 1.84), SW/SH: 0.62 (min 0.54/max 0.72), ah/SH: 0.41 (min 0.37/max 0.50).

**Figure 3.**
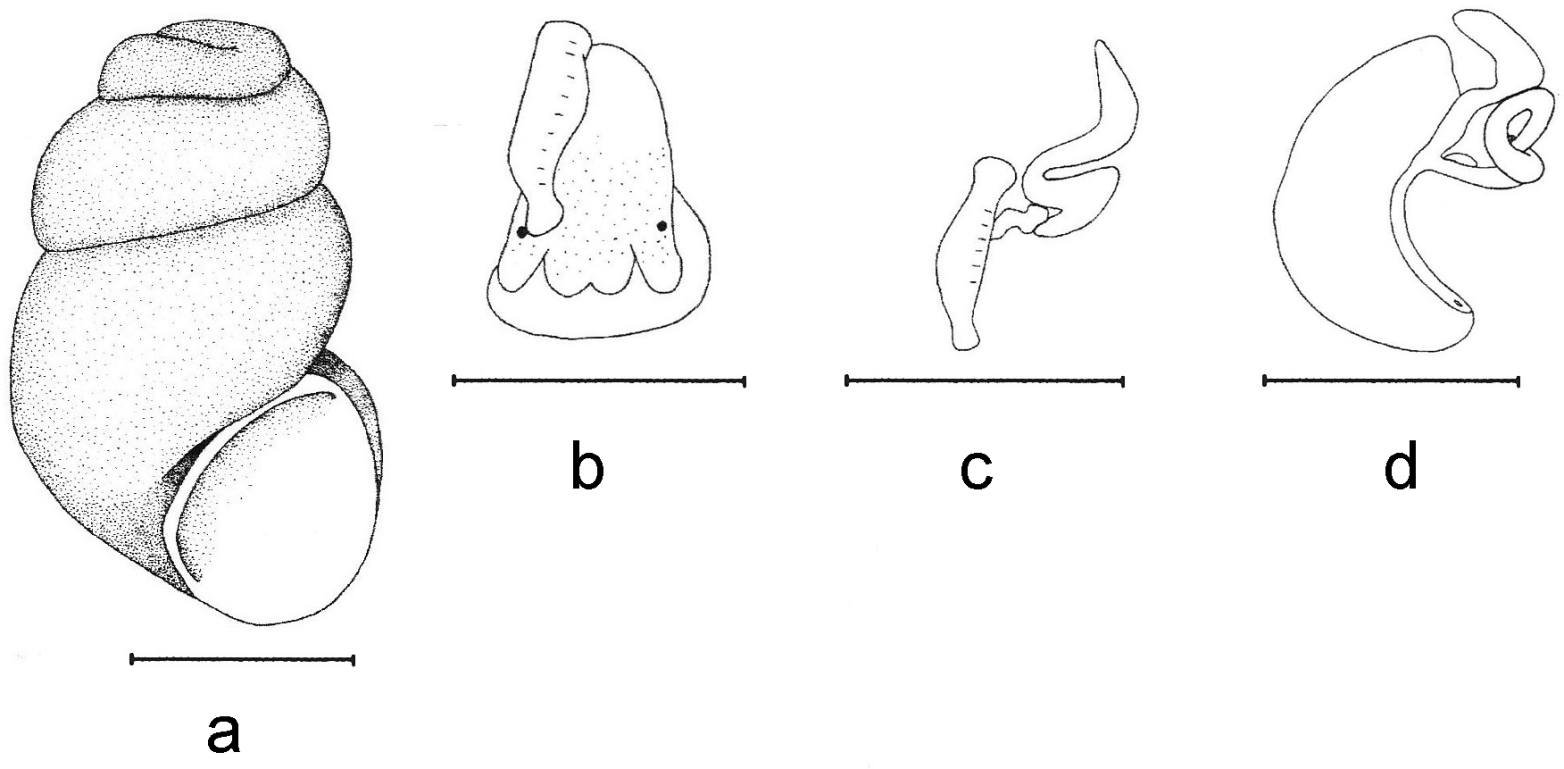
Shell and anatomy of *Bythinella istanbulensis* sp. n.: **a** shell **b** male head and penis in natural position **c** penis **d** female genitalia. Scale bars = 1 mm.

#### Differential diagnosis.

This new species is distinguished from other Turkish *Bythinella* species by its nearly cylindrical shell with broad and blunt apex. Small and attached rs_1_, unpigmented and light coloured pallial roof are the other key characters.

#### Habitat.

Specimens were collected from the stones in a small spring and its pond.

#### Remarks.

Based on shell characters only, Schütt (1965) misidentified *Bythinella* specimens from Belgrad Forest as *Bythinella opaca* (Frauenfeld, 1857), a species originally described from Carniola (Slovenia) and Italy. *Bythinella angelitae* was introduced as a replacement name for the preoccupied name *Paludinella opaca* Frauenfeld, 1857, a homonym of *Paludinella opaca* M. von Gallenstein, 1848, by Haase et al. (2007). The two closely related species *Bythinella angelitae* and *Bythinella opaca* can be morphologically separated only by radular dentition, and the geographic range of *Bythinella angelitae* is restricted to the border areas of Austria and Slovenia (Haase et al. 2007). Although they have been recorded in previous studies (Wagner 1941; Grossu 1956; Angelov 1960, 2000), *Bythinella austriaca* (Frauenfeld, 1857) and *Bythinella opaca* (M. von Gallenstein, 1848) have not been confirmed from the eastern Balkan countries in recent studies (Falniowski et al. 2009a, b). Therefore, there is a large distribution gap between the ranges of Central European taxa and that of *Bythinella istanbulensis* sp. n.

Despite the conchological similarities, the new species is anatomically distinct from the Central European species *Bythinella austriaca* and *Bythinella opaca*. While the lighter pallial roof colouration is also observed in *Bythinella opaca* (Glöer & Pešic, 2006), *Bythinella austriaca* has a dark pallial roof colouration. The new species differs from both of these taxa in having a very short and broad penial appendix (cf. long trumpet-shaped penial appendix in these species).

### Bythinella magdalenae

Taxon classificationAnimaliaLittorinimorphaBythinellidae

Yıldırım, Kebapçı & Bahadır Koca sp. n.

http://zoobank.org/669E7842-7985-473B-837B-C06917BF0962

Figs 4 , 6c

#### Holotype

(Coll. Yıldırım): SH 2,5 mm, SW 1,62 mm, ah 1,12 mm, aw 1,06 mm; TURKEY, Aydın, İncirliova, Karagözler Village, Karapınar spring above the village, 37°57,796'N, 27°49,375'E, leg. S. Bahadır Koca. Paratypes: 17 ex in Coll. Yıldırım, same data and locality as holotype; 18 ex in Coll. Yıldırım, TURKEY, Aydın, İncirliova, Karagözler Village, Çaycuk spring above the village, 37°57,829'N, 27°49,230'E, leg. S. Bahadır Koca.

#### Type locality.

Karapınar Spring, Karagözler Village, İncirliova, Aydın.

#### Etymology.

Named after the late Polish malacologist Magdalena Szarowska (1952–2013), who contributed greatly especially to the knowledge of the Balkan Truncatelloidea.

#### Description.

Shell ovate-conical and with 3–3,5 whorls. Apex truncated. Last whorl slowly increasing and broader than previous whorls, sutures not deep and periphery nearly flat. Aperture roundish oval. Umbilicus small, hollow shaped and covered by the lip. Operculum nucleus along left margin, oval shaped and with distinct growth lines.

Head having little amount of pigmentation, rather concentrated on the tips of the tentacles and around the mouth. Tentacles slightly longer than snout. Penis unpigmented, tapering towards the tip, tip not very pointed at its distal end, club-shaped. Tubular gland externally visible, thin, elongated and convoluted. Bursa copulatrix narrow and elongated, rs_1_ globular in shape. Central tooth of radula with 9 pointed cusps, 1 median and 4 each on sides; lateral margin without any cusps, but undulated.

Measurements: Karapınar Spring (n=18): SH: 2.55 mm (min 2.19/max 2.91), SW: 1.64 mm (min 1.37/max 1.78), SH/SW: 1.55 (min 1.42/max 1.72), SW/SH: 0.65 (min 0.58/max 0.70), ah/SH: 0.45 (min 0.44/max 0.46). Çaycuk Spring (n=18): SH: 2.41 mm (min 2.06/max 2.78), SW: 1.59 mm (min 1.31/max 1.87), SH/SW: 1.52 (min 1.37/max 1.66), SW/SH: 0.66 (min 0.64/max 0.67), ah/SH: 0.46 (min 0.46/max 0.48).

**Figure 4.**
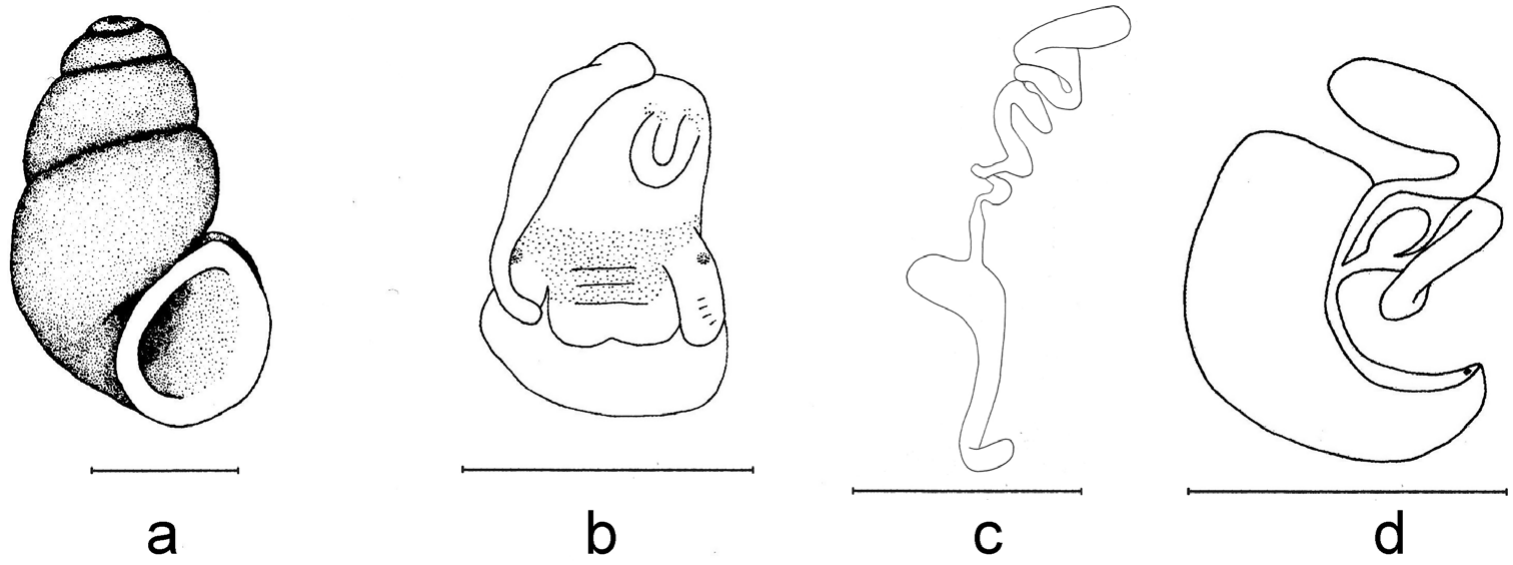
Shell and anatomy of *Bythinella magdalenae* sp. n.: **a** shell **b** male head and penis in natural position **c** penis **d** female genitalia. Scale bars = 1 mm.

#### Differential diagnosis.

*Bythinella magdalenae* sp. n. is distinguished from *Bythinella cosensis* Schütt 1980 (from Kos island) by the smaller number of shell whorls and the differing number of cusps on the central tooth of radula. It can be distinguished from other Turkish congeners by the flat periphery of the whorls and shallow sutures.

#### Habitat.

Specimens were collected from two small, closely proximal seepage springs.

#### Remarks.

Çaycuk Spring is very close to the type locality, thus indeed *Bythinella magdalenae* sp. n. can be considered a single spot endemic as in the case of other species known from Turkey.

### Bythinella wilkei

Taxon classificationAnimaliaLittorinimorphaBythinellidae

Yıldırım, Kebapçı & Bahadır Koca sp. n.

http://zoobank.org/3C7814DB-62E3-458B-9FB9-E4A026F339DD

Figs 5 , 6d

#### Holotype

(Coll. Yıldırım): SH 2.75 mm, SW 1.84 mm, ah 1.28 mm, aw 1.12 mm; TURKEY, Kocaeli, Maşukiye, spring along the road to Kartepe, 40°40.603'N, 30°08.605'E, leg. S. Bahadır Koca. Paratypes: 29 ex in Coll. Yıldırım, same data and locality as holotype.

#### Type locality.

Spring along the road to Kartepe, Maşukiye, Kocaeli.

#### Etymology.

Named after the malacologist Thomas Wilke (Justus Liebig University, Germany).

#### Description.

Shell oval conical, light brown, but appearing blackish due to encrustation; having 3–3.5 tumid whorls; last whorl slowly increasing and broader than previous whorls, sutures deep. Apex blunt. Aperture roundish oval, last whorl strongly descending towards aperture and aperture projected forward, lip in some individuals having a small protuberance in upper palatal margin. Umbilicus small, hollow or completely covered by the lip. Operculum ovate, translucent and with distinct growth lines.

Head having little pigmentation, pigment rather concentrated on bases of the tentacles and around mouth. Tentacles short and broad. Unpigmented penis tapering towards the tip, tip not pointed at its distal end; tubular gland thick and with two convolutions. Bursa copulatrix narrow and elongated, rs_1_ elongate. Central tooth of radula with 9 pointed cusps, 1 median and 4 each on sides; lateral margins lacking cusps, but undulated.

Measurements (n=22): SH: 2.80 mm (min 2.56/max 3.03), SW: 1.88 mm (min 1.56/max 2.19), SH/SW: 1.50 (min 1.31/max 1.68), SW/SH: 0.66 (min 0.60/max 0.76), ah/SH: 0.47 (min 0.41/max 0.51).

**Figure 5.**
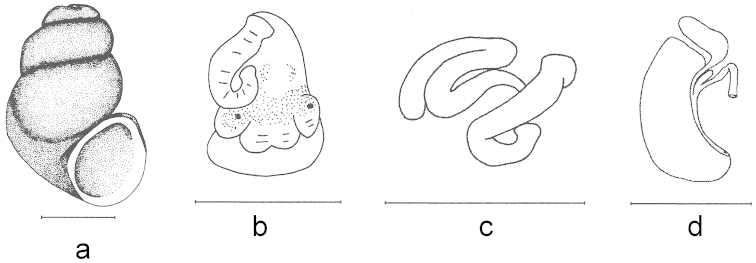
Shell and anatomy of *Bythinella wilkei* sp. n.: **a** shell **b** male head and penis in natural position **c** penis **d** female genitalia. Scale bars = 1 mm.

**Figure 6.**
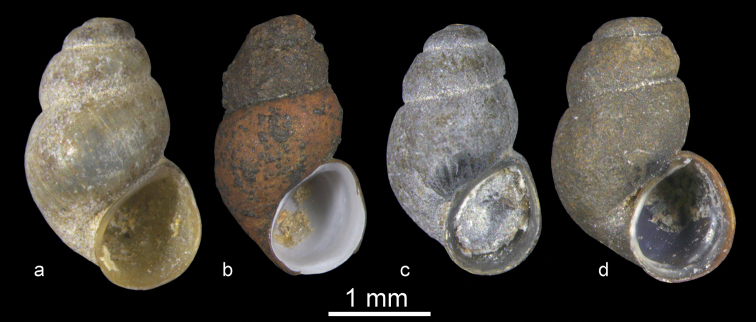
*Bythinella* species from western Anatolia: **a** *Bythinella anatolica* sp. n. **b** *Bythinella istanbulensis* sp. n. **c** *Bythinella magdalenae* sp. n. **d** *Bythinella wilkei* sp. n.

#### Differential diagnosis.

Excluding *Bythinella anatolica* sp. n. described from Manisa Province, *Bythinella wilkei* sp. n. has a larger shell than other Turkish congeners. The nearly circular aperture, which is strongly descending and projected forward, is also characteristic of the new species.

#### Habitat.

The specimens were collected from the outflow of a small spring having little vegetation and a gravel substrate.

#### Remarks.

The species is one of the two northernmost distributed congeners in Turkey. Unlike *Bythinella istanbulensis* sp. n. the new species is found in higher altitudes.

### Identification key to the *Bythinella* species from Turkey

**Table:** 

1	Shell having 3.5–4 whorls	**2**
–	Shell having 3–3.5 whorls	**4**
2	Shell elongated oval, aperture margins reflected	***Bythinella anatolica***
–	Shell nearly cylindrical, aperture margins not reflected	**3**
3	Pallial roof black, penis shorter than penial appendix	***Bythinella kazdaghensis***
–	Pallial roof light coloured, penis longer than penial appendix	***Bythinella istanbulensis***
4	Tubular gland thin, long and convoluted (more than 2 loops)	**5**
–	Tubular gland thick, short and less convoluted	***Bythinella wilkei***
5	Shell oval, SH<2.3 mm	***Bythinella occasiuncula***
–	Shell ovate-conic and usually SH>2.3 mm	**6**
6	Periphery almost straight, sutures shallow, tentacles longer than proboscis	***Bythinella magdalenae***
–	Periphery convex, sutures deep, tentacles shorter than proboscis	***Bythinella turca***

**Figure 7.**
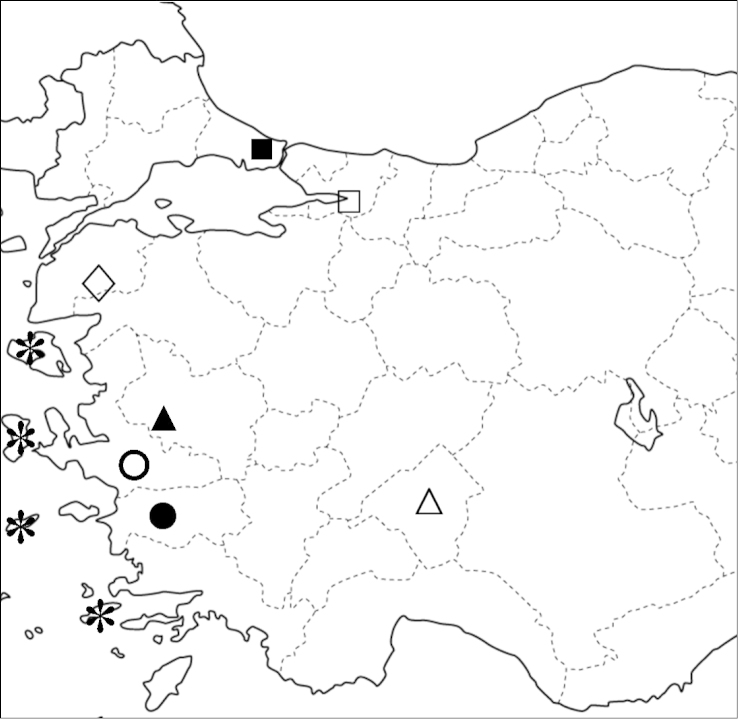
Map showing the locations of *Bythinella* species in Turkey and the Eastern Aegean Islands: *Bythinella anatolica* sp. n. (black triangle), *Bythinella istanbulensis* sp. n. (closed square), *Bythinella kazdaghensis* (diamond), *Bythinella kosensis* (asterix), *Bythinella magdalenae* sp. n. (closed circle), *Bythinella occasiuncula* (open circle), *Bythinella turca* (open triangle), *Bythinella wilkei* sp. n. (open square).

## Supplementary Material

XML Treatment for Bythinella

XML Treatment for Bythinella anatolica

XML Treatment for Bythinella istanbulensis

XML Treatment for Bythinella magdalenae

XML Treatment for Bythinella wilkei
